# A Study of Sex Differences in the Biological Pathways of Stress Regulation in Mice

**DOI:** 10.1111/cns.70433

**Published:** 2025-05-14

**Authors:** Yajun Qiao, Hanxi Chen, Juan Guo, Xingfang Zhang, Xinxin Liang, Lixin Wei, Qiannan Wang, Hongtao Bi, Tingting Gao

**Affiliations:** ^1^ Qinghai Provincial Key Laboratory of Tibetan Medicine Pharmacology and Safety Evaluation Northwest Institute of Plateau Biology, Chinese Academy of Science Xining China; ^2^ School of Psychology Chengdu Medical College Chengdu China; ^3^ University of Chinese Academy of Sciences Beijing China; ^4^ Medical College, Qinghai University Xining China; ^5^ CAS Key Laboratory of Tibetan Medicine Research Northwest Institute of Plateau Biology, Chinese Academy of Sciences Xining China; ^6^ Department of Psychiatry The People's Hospital of Jiangmen, Southern Medical University Jiangmen China

**Keywords:** behavior, brain–gut axis, depression, sex differences, stress

## Abstract

**Background:**

Stress is closely related to life, and it can also cause many mental disorders. However, there are significant sex differences in neuropsychiatric disorders associated with stress, particularly in depression, where the lifetime risk of depression in women is approximately twice that of men. However, the specific mechanism of this process has not been explained in detail.

**Methods:**

Chronic restraint stress (CRS) + chronic and unpredictable mild stress (CUMS) was used to simulate social stress, and behavioral experiments, HE staining of rectal and hippocampal pathological sections, detection of depression‐related biological indicators, analysis of intestinal flora diversity, and metabolomics analysis of hippocampal and intestinal contents were performed.

**Results:**

The results showed that stress induced anxiety‐like behavior in female mice and depression‐like behavior in male mice. Sex differences in behavior may be related to monoamine neurotransmitters, hyperactivity of HPA axis, inflammatory factors, gut microbiota, and brain–gut metabolism. It is worth noting that stress caused opposite trends in DA (dopamine) levels, abundance of f‐lactobaciliaceae, and levels of metabolites (1, 2‐distearoyl‐SN‐glycero‐3‐phosphocholine) and PC(20:5(5Z,8Z,11Z,14Z,17Z)/20:1(11Z)) in male and female mice.

**Conclusion:**

The difference in neurotransmitter levels, the disorder of gut microbiota, and the abnormal brain and gut metabolism may lead to the gender difference in stress behavior.

## Graphical abstract

Stress induced anxiety‐like behavior in female mice and depression‐like behavior in male mice. Sex differences in behavior may be related to monoamine neurotransmitters, inflammatory factors, gut microbiota, and brain–gut metabolism. Notably, stress caused opposite trends in DA levels, *f‐Lactobaciliaceae* abundance, and metabolites (G‐DSGP and PC‐20) levels in male and female mice, and the metabolic pathway was the arachidonic acid metabolism pathway. These findings may in the future provide evidence for gender differences in stress‐induced mental illness. Abbreviations: DA, Dopamine; DSGP, 1,2‐distearoyl‐sn‐glycero‐3‐phosphocholine; FC, Female Control; FM, Female Model; MC, Male Control; MM, Male Model; PC‐20, PC(20:5(5Z,8Z,11Z,14Z,17Z)/20:1(11Z).

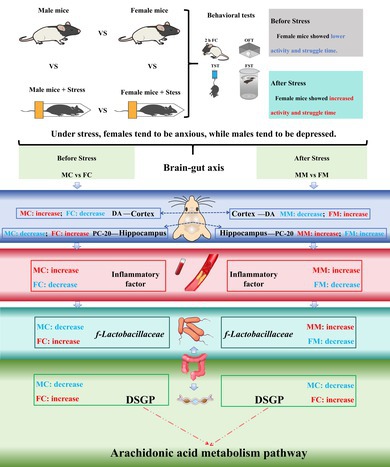

**Abbreviations**
2 h‐FC: 2 hour‐food consumption
5‐HT: 5‐hydroxytryptamine
AA: arachidonic acid
ACTH: adrenocorticotropic hormone
ARRIVE: Animal Research: Reporting of In Vivo Experiments
CALP: calprotectin
CE: collision energy
CMD: center movement distance
CORT: cortisol
COVID‐19: Corona virus disease‐2019
CRH: corticotropin releasing hormone
CRS: chronic‐restraint stress
CRT: center residence time
CUMS: chronic unpredictable mild stress
CUR: ncurtain gas
DA: dopamine
DMs: different metabolites
DP: declustering potential
FC: female control
FM: female model
FST: forced swim test
G‐DSGP: gut 1,2‐distearoyl‐sn‐glycero‐3‐phosphocholine
GR: glucocorticoid receptor
H‐DSGP: hippocampus‐1,2‐Dioleoyl‐sn‐Glycero‐3‐Phosphocholine
HILIC: hydrophilic interaction liquid chromatography
HOSGP: 1‐hexadecanoyl‐2‐octadecanoyl‐sn‐glycero‐3‐phosphocholine
HPA: hypothalamic–pituitary–adrenal
IDA: information‐dependent acquisition
IL‐10: interleukin‐10
IL‐4: interleukin‐4
IL‐6: interleukin‐6
ISVF: ion spray voltage floating
KM: Kunming
LDA: linear discriminant analysis
Lefse: linear discriminant analysis effect size
LEP: leptin
LPS: lipopolysaccharide
MC: male control
MD: movement distance
MM: male model
MR: Mineralocorticoid receptor
MT: movement time
NE: norepinephrine
OFT: open field test
PC: phosphatidylcholine
PC‐20: PC(20:5(5Z, 8Z, 11Z, 14Z, 17Z)/20:1(11Z))
PCA: principal component analysis
RT: rest time
TNFα: tumor necrosis factor‐α
TSH: thyroid‐stimulating hormone
TST: tail suspension test

## 1 Introduction

Stress occurs throughout our lives, and stress causes many mental illnesses, including major depression, bipolar disorder, and posttraumatic stress disorder [1]. Currently, the most studied stress‐induced mental illness is depression. Depression affects approximately 350 million people worldwide, and the 2019–2023 COVID‐19 (Corona Virus Disease 2019) pandemic led to a rapid increase in the number of individuals with depression, anxiety, and other mental disorders [2, 3, 4]. However, the incidence of neuropsychiatric diseases related to disorders caused by stress differs between men and women [5]. Studies have shown a significant sex difference in depression, with women being more prone to depression and having a lifetime risk of depression approximately twice that of men [6, 7]. Although psychological and cultural factors unique to women contribute to sex differences in disease, there is evidence that biological factors also play a key role. Biological factors may contribute to the different manifestations of depression in men and women [8, 9].

At present, the neurobiological pathogenesis of depression mainly involves the monoamine neurotransmitter pathway [10], the stress‐responsive HPA (hypothalamic–pituitary–adrenal) axis [11], neuroinflammation [12], and the microbiome–gut–brain axis [13, 14]. Interestingly, both serotonin and dopamine are present at higher concentrations in women [15], and we know that both of these neurotransmitters are associated with depression and anxiety‐like symptoms. However, sex differences are reflected not only in the concentrations of monoamine neurotransmitters but also in the activation pathway of the HPA axis. Teo et al. reported that the roles of GR (Glucocorticoid receptor) and MR (Mineralocorticoid receptor) in the HPA axis may differ between males and females during depression, with increased HPA activity and upregulated MR expression levels in male mice, while the opposite is true in female mice [16]. In addition, there are sex differences in the gut microbiota [17, 18], and Li et al. reported that the depressive behavior of premenopausal women may be related to the “eating” of estradiol by 3β‐hydroxysteroid dehydrogenase produced by gut microbiota [19]. Therefore, we hypothesize that sex differences in depression‐like behaviors caused by stress may be related to differences in monoamine neurotransmitter levels, stress hormone secretion, and gut microbiota. However, the causes of stress differences between the sexes are currently summarized in different levels of hormone metabolism [5, 20, 21]. Most animal studies on the differences between male and female stress have focused on behavior and neural circuits [22, 23], and a few studies have observed differences in brain regions and gut metabolism in the process of male and female stress [19, 24], and there is a lack of studies that systematically combine brain and gut metabolism to analyze the differences between male and female stress. Since the brain–gut axis plays an important role in the stress process, we hypothesize that there are sex‐specific stress differences in the brain and gut, so this study will be designed to test around this hypothesis. We explored the differential effects of stress on male and female mice through the evaluation of common biological indicators of depression (monoamine neurotransmitters, appetite hormones, HPA axis core indices, cytokines), intestinal flora, brain‐intestinal pathological injury, and brain‐intestinal metabolism.

## 2 Materials and Methods

### 2.1 Animals

Kunming (KM) mice (male/female) aged 5–6 weeks were purchased from SiPeiFu (Beijing) Biotechnology Co. Ltd. (license number: SCXK [Jing] 2019‐0010). The mice were housed in standard cages containing wood shavings placed in a room with a carefully controlled ambient temperature (22°C ± 1°C) and artificial lighting from 7:00 am to 19:00 pm and were given standard laboratory chow and distilled water ad libitum. All the animal experiments were in compliance with the ARRIVE (Animal Research: Reporting of In Vivo Experiments) guidelines, were carried out in strict accordance with the National Institutes of Health Guide for the Care and Use of Laboratory Animals (NIH Publication No. 8023, revised 1978), and were approved by the Committee of the Northwest Plateau Institute of Biology, CAS, for animal experiments (allowance number NWIPB20171106‐01).

### 2.2 Materials

Enzyme‐linked immunosorbent assay (ELISA) kits for 5‐hydroxytryptamine (5‐HT), norepinephrine (NE), dopamine (DA), ghrelin, leptin (LEP), thyroid stimulating hormone (TSH), cortisol (CORT), adrenocorticotropic hormone (ACTH), corticotropin releasing hormone (CRH), tumor necrosis factor α (TNFα), interleukin (IL)‐4, IL‐6, IL‐10, calprotectin (CALP), and lipopolysaccharide (LPS) were obtained from the Shanghai Jinglai Bioengineering Institute (Shanghai, China). RNAiso Plus reagent (item No. 9109, Takara Biotechnology Co. Ltd), trichloromethane (item no. 10006818), isopropyl alcohol (item no.: 80109218), and anhydrous ethanol (item no. 10009218) were purchased from Sinopsin Chemical Reagent Co. Ltd. RT Easy TM II reverse transcription kit (item no. RT‐01022/01023, FOREGENE), SYBR qPCR SuperMix plus kit (item no. M00041, Sichuan Lanyun Biotechnology Co. Ltd.), and primers were purchased from Chengdu Qingke Biological Co. Ltd (see Appendix S1 for details).

### 2.3 Stress Model Establishment and Animal Grouping

**FIGURE 1:**
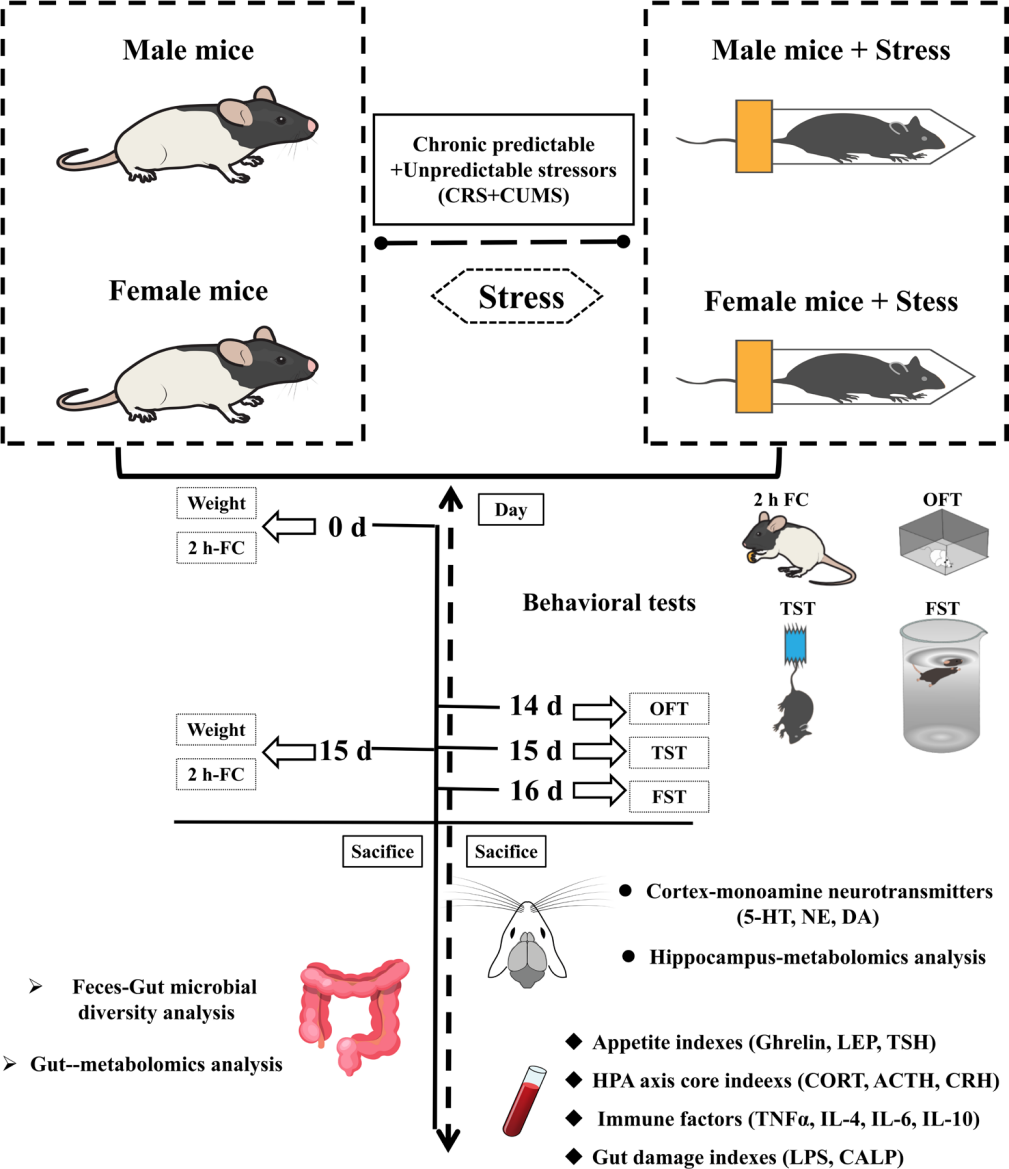
Flow chart of the test.

After 1 week of acclimation, the mice (*n* = 32) were randomly divided into the following four groups with eight mice in each group: (1) male control (MC), (2) male model (MM), (3) female control (FC), and (4) female model (FM) groups. In addition, all mice except those in the control groups were exposed to chronic predictable and unpredictable stressors, including CRS and CUMS, for 14 days. CUMS exposure consisted of placing the mice in tubes from 8:30 am to 14:30 pm each day [25]. The CUMS conditions were as follows: 5 min of heat stress at 45°C, 2 min of cold stress at 10°C, 2 min of reciprocating sway, 24 h in 45°‐tilted cages in a humid environment, 24 h of food deprivation, 24 h of water deprivation, and 24 h of day‐and‐night reversal. Each condition was randomly arranged and performed two times per day [26, 27]. The test process is shown in Figure 1.

### 2.4 Weight and 2‐h Food Consumption (2‐h FC) Test

Weight and 2‐h FC measurements were performed both before and after stress stimulation (0, 8, and 15 days). FC was measured at 21:00 on the day before the fast began. The mice were then fasted for 22 h. At 19:30 the next day, the mice were placed in individual cages and given food and water. After 2 h, the food was weighed, and 2‐h FC was calculated [27, 28].

### 2.5 Behavioral Test

Behavioral tests, including the open field test (OFT), the tail suspension test (TST), and the forced swim test (FST), were performed after 2 weeks of stress. According to the damage intensity of behavioral tests on mice, from weak to strong, behavioral tests were performed every day (14 days: OFT; 15 days: TST; 16 days: FST). The OFT was performed following the procedures described by Choleris et al. [29]. The TST was performed following the procedures described by Andreasen and Redrobe [30]. Finally, the FST was carried out following the procedures described by Porsolt et al. [31].

### 2.6 Evaluation of Monoamine Neurotransmitter Levels, Appetite Indices, Immune Factors, and Gut Damage Indices

Enzyme‐linked immunosorbent assays (ELISAs) were used to measure the levels of monoamine neurotransmitters, appetite indices, immune factors, and gut damage indices. After behavioral test completion, mouse blood was collected through the retroorbital venous bundle and placed in a collection tube containing an appropriate amount of anticoagulant. The collection vessels were inverted repeatedly so that the anticoagulant and blood were fully mixed, and plasma was obtained by centrifugation at 4°C and 1509.3 ×g for 10 min. Immediately after blood collection, the animals were euthanized, and the cortex and hypothalamus were collected and mixed with phosphate buffered saline (pH 7.4). The tissues were then homogenized using a tissue homogenizer (frequency: 60 Hz, rotation rate: 1800 beats/min, duration: 2 min), and the supernatants were centrifuged at 4°C and 4192.5 ×g. The cortex, hypothalamus, and serum samples of the mice were tested via ELISA according to the instructions of the kit manufacturer. An enzyme‐labeling apparatus (Enspire 2300, PerkinElmer, USA) was used. 5‐HT, NE, DA, ghrelin, LPE, TSH, TNFα, IL‐4, IL‐6, IL‐10, CALP, and LPS were detected at 450 nm. All ELISA kits were purchased from Shanghai Jianglai Biotechnology Co. Ltd., China.

### 2.7 Hematoxylin and Eosin (H&E) Staining of Brain and Gut Tissue

After the behavioral test completion, blood was extracted from the necks of the mice, and the brain and rectal tissues were quickly removed from the mice and placed on ice plates. Whole brain and rectal tissues were fixed in 4% paraformaldehyde solution for 24 h, paraffin‐embedded, and subjected to sagittal sectioning (with a slice thickness of 5 μm). H&E staining was performed, and pathological changes in the hippocampus and rectum were observed under a microscope (Leica DM 1000).

### 2.8 16S rDNA Analysis

The microbiome was used to reflect changes in gut microbiota. After completion of the behavioral tests, the mice were anesthetized with an isoflurane anesthesia machine (RWD Life Sciences Co. Ltd., Shenzhen, China), and the fresh rectal contents of the mice were collected, frozen quickly in liquid nitrogen, and placed in a freezer at −80°C for subsequent analysis [32]. DNA was extracted from each rectal content sample (*n* = 10 per group) using the Mag‐Bind Soil DNA Kit (Omega Biotek, United States) following the manufacturer's protocol. The purity and concentration of DNA were measured by 1% agarose gel electrophoresis. The V3–V4 hypervariable region of the 16S rDNA gene was subsequently PCR amplified using the universal primers 338F and 806R. The PCR amplicons were purified, quantified, and sequenced on the Illumina NovaSeq 600 platform. Clean data were obtained by FLASH software by merging paired‐end reads. The UPARSE software package using the UPARSE‐OTU and UPARSE‐OTUref algorithms was used for operational taxonomic unit (OTU) clustering and species annotation. Then, graphical representations of the relative abundance of bacteria from phylum to species were generated using a Krona chart.

### 2.9 Metabolite Analysis

Metabolomics was used to analyze the differentially abundant metabolites in the gut and hippocampus. After 16S rDNA sequencing was completed, the remaining samples were subjected to metabolome analysis. The analysis was performed using a UHPLC (1290 Infinity LC, Agilent Technologies) with an HILIC chromatographic column coupled to a quadrupole time‐of‐flight mass spectrometer (AB Sciex TripleTOF 6600) [33]. For HILIC separation, the samples were analyzed using a 2.1 mm × 100 mm ACQUITY UPLC BEH Amide 1.7 μm column (Waters, Ireland). In both the ESI‐positive and ESI‐negative modes, the mobile phase contained A = 25 mM ammonium acetate, 25 mM ammonium hydroxide in water, and B = acetonitrile. The gradient was 95% B for 0.5 min and was linearly reduced to 65% in 6.5 min, reduced to 40% in 1 min, maintained for 1 min, and then increased to 95% in 0.1 min, with a 3 min re‐equilibration period.

The ESI source conditions were set as follows: ion source gas 1 (Gas1), 60; ion source gas 2 (Gas2), 60; curtain gas (CUR), 30; source temperature, 600°C; and ion spray voltage floating (ISVF), ±5500 V. In MS‐only acquisition, the instrument was set to acquire signals over the *m*/*z* range of 60–1000 Da, and the accumulation time for the TOF MS scan was set at 0.20 s/spectra. In auto MS/MS acquisition, the instrument was set to acquire signals over the *m*/*z* range of 25–1000 Da, and the accumulation time for the product ion scan was set at 0.05 s/spectra. The product ion scan was acquired using information‐dependent acquisition (IDA) in high‐sensitivity mode. The parameters were set as follows: collision energy (CE) was fixed at 35 V ± 15 eV; declustering potential (DP) was set at 60 V (+) and −60 V (−); isotopes within 4 Da were excluded; and the number of candidate ions for monitoring each cycle was 10. The raw MS data were converted to MzXML files using ProteoWizard MSConvert before being imported into freely available XCMS software for peak grouping, retention time correction, and peak area extraction.

### 2.10 Quantitative Polymerase Chain Reaction (q‐PCR)

The hippocampus was removed and melted on ice. One milliliter of Trizol was absorbed into a 1.5 mL RNase‐free EP tube, and about 20 mg of the sample was cut into the EP tube for RNA extraction. The OD of RNA in the hippocampus was detected by scandrop100. FOREGENE reverse transcription kit (RT Easy TM II) was used for the reverse transcription operation (20 μL reaction system). After the reaction, cDNA was obtained and stored at −80°C for later use. Primers were designed for β‐actin, cyp2j11, cyp2c66, cyp2c37 (primers were designed and synthesized in primer5 or BD according to sequences retrieved from NCBI) (Tables S1 and S2); All components were configured according to the q‐PCR reaction system (SYBR q‐PCR SuperMix plus), centrifuged on a PCR plate centrifuge at 6037.2 ×g at 4°C for 30 s, and then amplified in a quantitative PCR instrument according to the above procedures. With β‐actin as the internal parameter, the relative gene expression of each sample was calculated by the ΔΔCT method (2^−ΔΔCT^) [34]. For details of the test (Figures S1 and S2), see Appendix S1.

### 2.11 Statistical Analysis

All data in this paper are expressed as mean (± standard deviation), and SPSS 22.0 software was used for data analysis. This experiment was tested for normality using the Shapiro–Wilk or Kolmogorov–Smirnov tests, and the data followed a normal distribution. When comparing more than two groups, data with normal distribution were analyzed using one‐way analysis of variance (ANOVA) and Tukey post hoc test, while data with nonnormal distribution were analyzed using Kruskal–Wallis test. In addition, Spearman and RDA (Redundancy Analysis) were used for association and dimensionality reduction analysis. *p* < 0.05 was considered statistically significant. Data were visualized using Origin 2022 software.

## 3 Results

### 3.1 Effects of Stress on Body Weight and 2‐h FC of Mice

**FIGURE 2:**
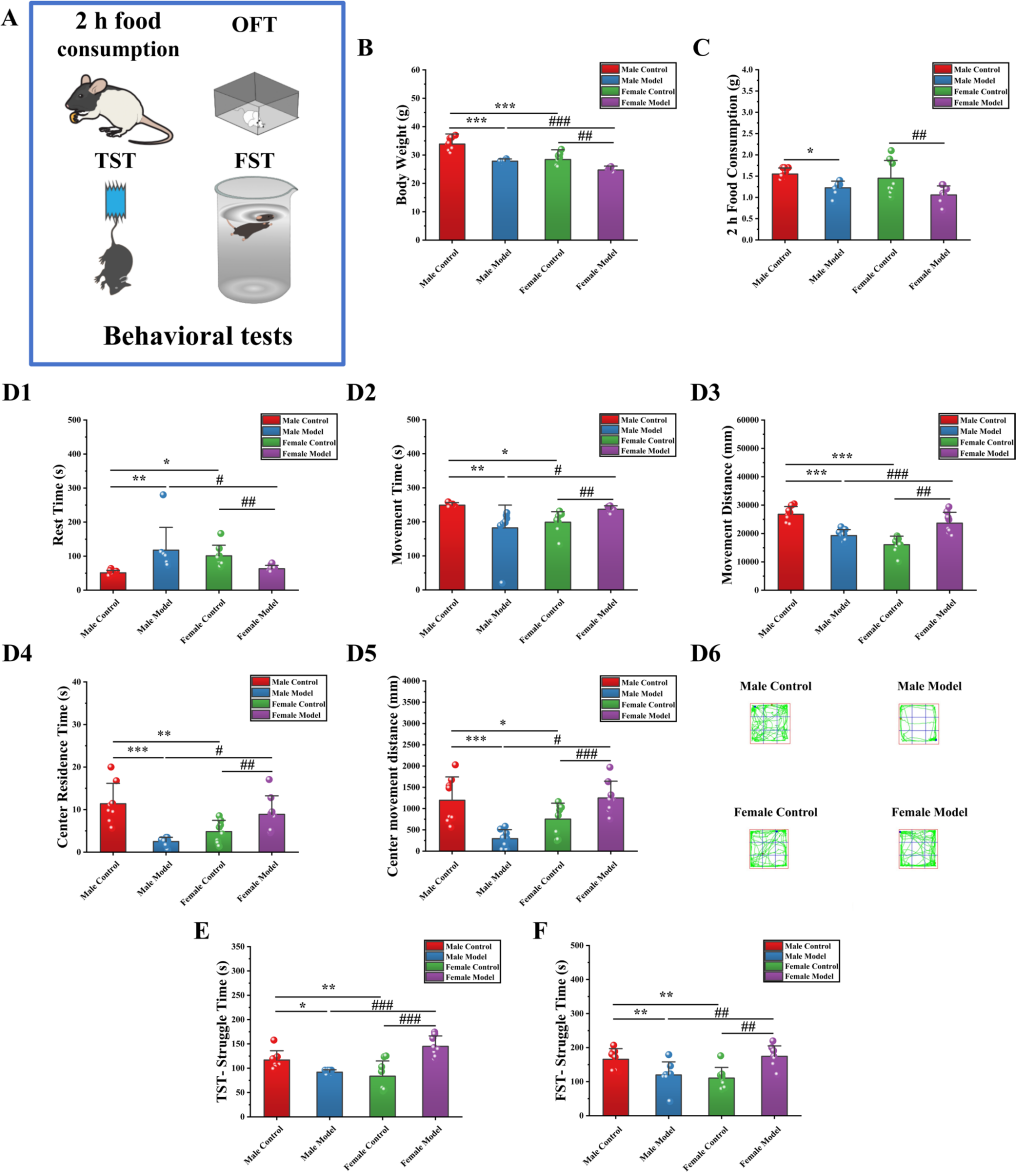
Effects of stress on behavior in mice. (A) Behavioral tests, (B) body weight, (C) 2‐h food consumption, (D) OFT, (E) TST, (F) FST. The data are shown as the means ± SDs (*n* = 8). *p < 0.05, **p < 0.01, and ***p < 0.001 denote significant differences from the MC group. ^#^ *p* < 0.05, ^##^ *p* < 0.01, and ^###^ *p* < 0.001 denote significant differences from the FM group.

After 14 days of stress, the body weights of the mice in each group were measured, as shown in Figure 2B. Compared with that of mice in the MC group, the body weight of mice in the MM group was significantly decreased by 17.81% (*p* < 0.001). Compared with that of mice in the FC group, the body weight of mice in the FM group was significantly decreased by 12.97% (*p* < 0.001). Compared with that of mice in the MC group, the body weight of mice in the FC group was significantly decreased by 16.03% (*p* < 0.001), and the body weight of mice in the FM group was significantly decreased by 11.08% (*p* < 0.001) compared with that of mice in the MM group. After 14 days of stress, the food consumption of the mice in each group was measured, as shown in Figure 2C. Compared with that of mice in the MC group, the 2‐h FC of mice in the MM group was significantly decreased by 20.97% (*p* < 0.05). Compared with that of mice in the FC group, the 2‐h food consumption of mice in the FM group was significantly decreased by 27.09% (*p* < 0.01). Compared with that of mice in the MC group, the 2‐h food consumption of mice in the FC group was decreased by 6.45%, and the 2‐h food consumption of mice in the FM group was decreased by 11.08% compared with that of mice in the MM group.

### 3.2 Effects of Stress on the Behavior of Mice in the OFT

As shown in Figure 2D1–D3, compared with that of mice in the MC group, the rest time (RT) of mice in the MM group was significantly increased by 130.46% (*p* < 0.01), and the movement time (MT: −26.77%, *p* < 0.01) and movement distance (MD: −28.00%, *p* < 0.001) were significantly decreased. Compared with those in the FC group, the RT of mice in the FM group was significantly decreased by 37.80% (*p* < 0.05), and the MT (19.24%, *p* < 0.05) and MD (47.34%, *p* < 0.001) were significantly increased. Compared with those in the MC group, the RT of mice in the FC group was significantly increased by 98.08% (*p* < 0.05), and the MT (−20.13%, *p* < 0.05) and MD (−40.09%, *p* < 0.001) were significantly decreased. Compared with those in the MM group, the RT of mice in the MM group was significantly decreased by 46.54% (*p* < 0.05), and the MT (30.06%, *p* < 0.01) and MD (22.60%, *p* < 0.01) were significantly increased.

As shown in Figure 2D4,D5, the center residence time (CRT: −78.05%, *p* < 0.001) and center movement distance (CMD: −75.42%, *p* < 0.001) of mice in the MM group were significantly decreased compared with those of mice in the MC group. Compared with those in the FC group, the CRT (83.89%, *p* < 0.001) and CMD (66.08731192%, *p* < 0.001) were significantly increased in the FM group. Compared with those of mice in the MC group, the CRT level (57.46%, *p* < 0.01) and CMD (36.99%, *p* < 0.05) of mice in the FC group were significantly increased. Compared with those of mice in the MM group, the CRT level (256.42%, *p* < 0.01) and CMD (325.72%, *p* < 0.001) of mice in the FM group were significantly increased. As shown in Figure 2D6, the activity level of the mice in the MC, FC, and FM groups was high, while the activity level of the mice in the MM group was low, and the activity range tended to remain in the surrounding area.

### 3.3 Effects of Stress on the Behavior of Mice in the TST and FST

The results of the detection of struggling behaviors are shown in Figure 2E,F. Compared with that of mice in the MC group, the ST (struggle time) of mice in the MM group was significantly decreased (TST: −21.46%, *p* < 0.05; FST: −27.89%, *p* < 0.01). Compared with that of the mice in the FC group, the ST of mice in the FM group was significantly increased (TST: 73.39%, *p* < 0.001; FST: 57.89%, *p* < 0.01). Compared with that of mice in the MC group, the ST of mice in the FC group was significantly increased (TST: 28.44%, *p* < 0.01; FST: 33.38%, *p* < 0.01). Compared with that of the mice in the MM group, the ST of mice in the FM group was significantly increased (TST: 57.97%, *p* < 0.001; FST: 45.86%, *p* < 0.01).

### 3.4 Effects of Stress on the Biological Indices of Mice

#### 3.4.1 Effects of Stress on Monoamine Neurotransmitter Levels in Mice

**FIGURE 3:**
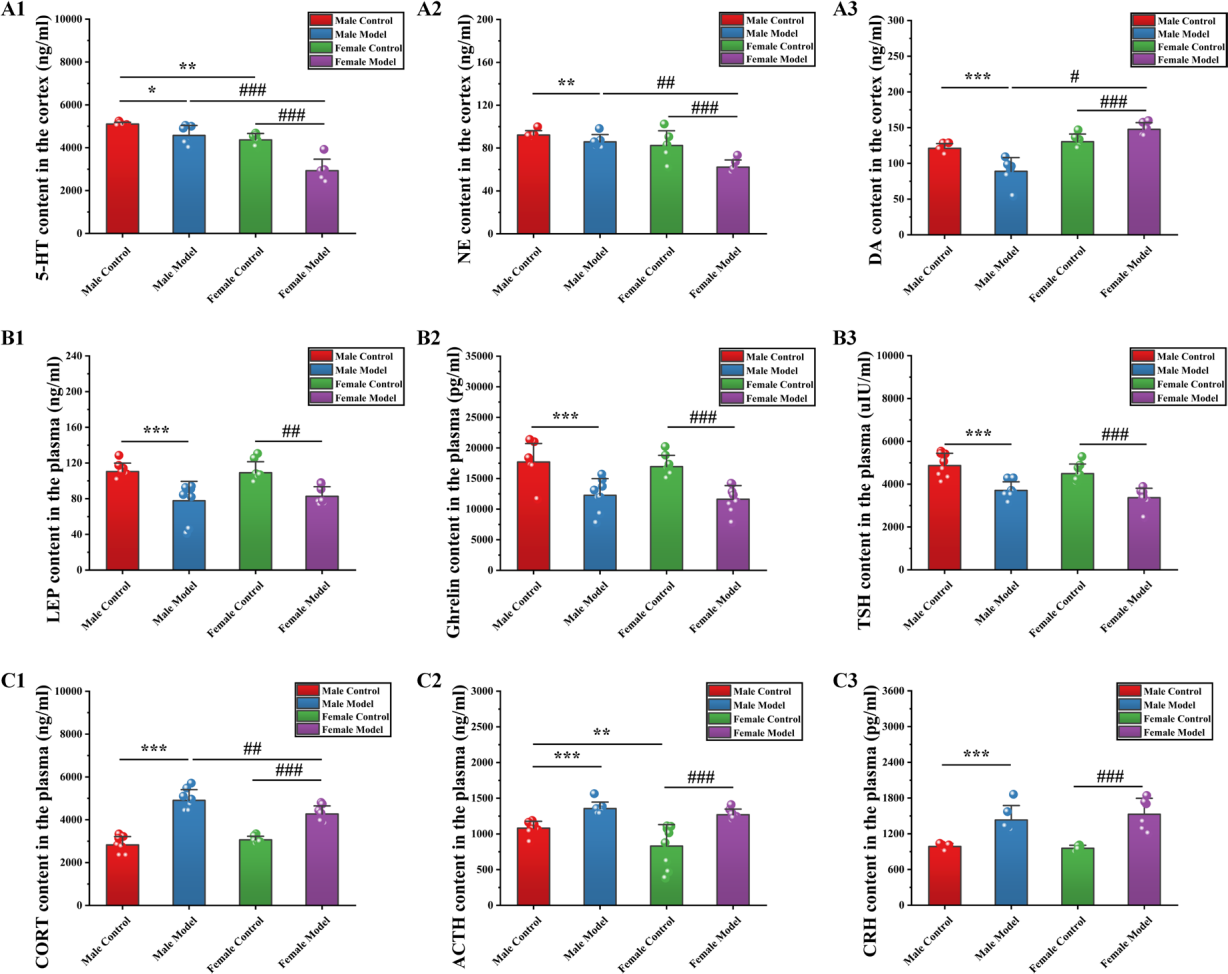
Effects of stress on (A) monoamine neurotransmitter, (B) appetite hormone, and (C) HPA core index levels in mice. The data are shown as the means ± SDs (monoamine neurotransmitter and CRH: *N* = 6; other indicators: *N* = 8). *p < 0.05, **p < 0.01, and ***p < 0.001 denote significant differences from the MC group. ^#^ *p* < 0.05, ^##^ *p* < 0.01, and ^###^ *p* < 0.001 denote significant differences from the FM group.

The results of monoamine neurotransmitter level measurements are shown in Figure 3A. Compared with those of mice in the MC group, the levels of 5‐HT (−10.42%, *p* < 0.05), NE (−6.93%, *p* < 0.01), and DA (−26.55%, *p* < 0.001) of mice in the MM group were significantly decreased. Compared with those of mice in the FC group, the levels of DA (13.37%, *p* < 0.05) of mice in the FM group were significantly increased, and the levels of 5‐HT (−32.93%, *p* < 0.001) and NE (−24.53%, *p* < 0.001) in the FM group were significantly decreased. Compared with those of mice in the MC group, the levels of DA (7.09%) of mice in the FC group were increased, and the levels of 5‐HT (−16.88%, *p* < 0.01) and NE (−11.79%) were significantly decreased. Compared with those of mice in the MM group, the levels of DA (66.13%, *p* < 0.001) were significantly increased in mice in the FM group, while the levels of 5‐HT (−35.94%, *p* < 0.001) and NE (−27.46%, *p* < 0.01) were significantly decreased in mice in the FM group.

#### 3.4.2 Effects of Stress on the Levels of Appetite Hormones in Mice

As shown in Figure 3B, the LEP (−29.57%, *p* < 0.001), ghrelin (−30.75%, *p* < 0.01), and TSH (−23.83%, *p* < 0.001) levels of mice in the MM group were significantly decreased compared with those of mice in the MC group. Compared with those of mice in the FC group, the LEP (−24.21%, *p* < 0.01), ghrelin (−31.43%, *p* < 0.001), and TSH (−24.98%, *p* < 0.001) levels of mice in the FM group were significantly decreased. Compared with those of mice in the MC group, the LEP (−1.28%), ghrelin (−4.29%), and TSH (−7.78%) levels of mice in the FC group were significantly decreased. Compared with those of mice in the MM group, the LEP level of mice in the FM group was increased by 6.24%, and the ghrelin (−5.23%) and TSH (−9.16%) levels were decreased.

#### 3.4.3 Effects of Stress on the HPA Axis in Mice

As shown in Figure 3C, the levels of CORT (73.68%, *p* < 0.001), ACTH (25.41%, *p* < 0.01), and CRH (45.18%, *p* < 0.001) of mice in the MM group were significantly increased compared with those of mice in the MC group. Compared with those of mice in the FC group, the levels of CORT (39.30%, *p* < 0.001), ACTH (52.82%, *p* < 0.001), and CRH (59.54%, *p* < 0.001) of mice in the FM group were significantly increased. Compared with those of mice in the MC group, the CORT level of mice in the FC group was increased by 8.47%, and the ACTH (−23.15%, *p* < 0.01) and CRH (−2.96%, *p* < 0.001) levels were significantly decreased. Compared with those of mice in the MM group, the CRH level of mice in the FM group was increased by 6.64%, and the CORT (−13.00%, *p* < 0.01) and ACTH (−6.36%, *p* < 0.01) levels were significantly decreased.

#### 3.4.4 Effects of Stress on Cytokine Levels in Mice

**FIGURE 4:**
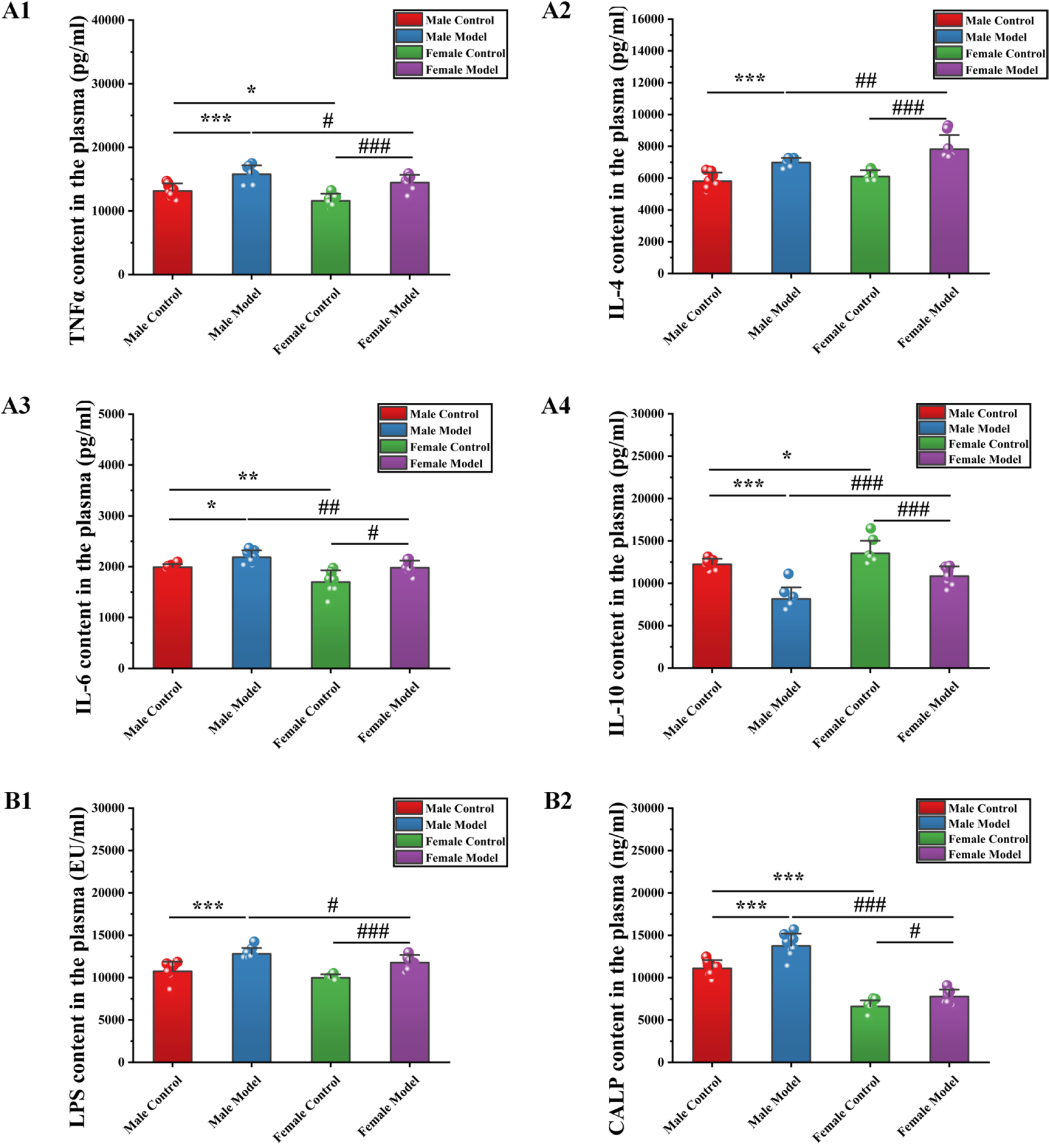
Effects of stress on (A) cytokine and (B) gut injury index levels in mice. The data are shown as the means ± SDs (*n* = 8). *p < 0.05, **p < 0.01, and ***p < 0.001 denote significant differences from the MC group. ^#^ *p* < 0.05, ^##^ *p* < 0.01, and ^###^ *p* < 0.001 denote significant differences from the FM group.

As shown in Figure 4A, compared with those of mice in the MC group, the levels of TNFα (19.99%, *p* < 0.001), IL‐4 (20.20%, *p* < 0.001), and IL‐6 (9.70%, *p* < 0.05) of mice in the MM group were significantly increased, whereas the level of IL‐10 was significantly decreased by 33.43% (*p* < 0.001). Compared with those of mice in the FC group, the levels of TNFα (24.71%, *p* < 0.001), IL‐4 (28.23%, *p* < 0.001), and IL‐6 (16.39%, *p* < 0.01) were significantly increased in mice in the FM group, whereas the level of IL‐10 was significantly decreased by 19.83% (*p* < 0.001). Compared with those of mice in the MC group, the levels of IL‐4 (4.95%) and IL‐10 (10.64%, *p* < 0.001) of mice in the FC group were significantly increased, whereas the levels of TNFα (−11.68%, *p* < 0.05) and IL‐6 (−14.74%, *p* < 0.01) were significantly decreased. Compared with those of mice in the MM group, the levels of IL‐4 (11.96%, *p* < 0.01) and IL‐10 (33.24%, *p* < 0.001) in mice in the FC group were significantly increased, whereas the levels of TNFα (−8.22%, *p* < 0.05) and IL‐6 (−9.55%, *p* < 0.01) were significantly decreased.

#### 3.4.5 Effects of Stress on the Levels of Gut Injury Indices in Mice

The results of gut injury index‐level measurements are shown in Figure 4B. Compared with those of mice in the MC group, the levels of LPS (19.09%, *p* < 0.001) and CALP (23.84%, *p* < 0.001) of mice in the MM group were significantly increased. Compared with those of mice in the FC group, the levels of LPS (18.06%, *p* < 0.001) and CALP (17.44%, *p* < 0.05) of mice in the FM group were significantly increased. Compared with those of mice in the MC group, the levels of LPS (−7.20%) and CALP (−40.54%, *p* < 0.001) of mice in the FC group were significantly decreased. Compared with those of mice in the MM group, the levels of LPS (−43.61%, *p* < 0.001) and CALP (−8.00%, *p* < 0.05) of mice in the FM group were significantly decreased.

### 3.5 Effects of Stress on Neurons in Hippocampal Subregions of Mice

**FIGURE 5:**
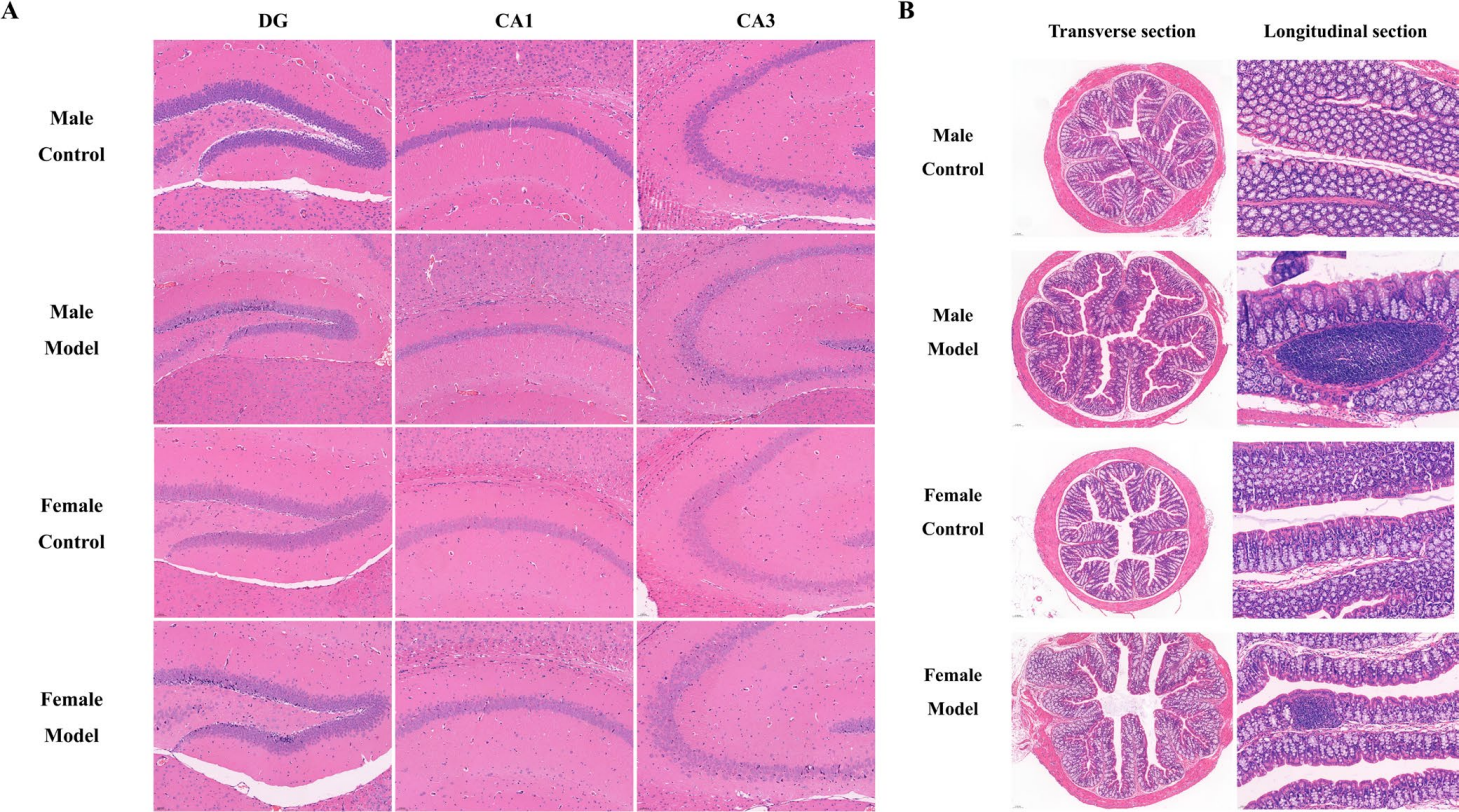
Effects of stress on (A) the hippocampal subregion and (B) rectal tissue in mice (H&E, *n* = 2; (A) bar = 50 nm; (B) Transverse section, bar = 100 nm; longitudinal section, bar = 50 nm).

The findings of pathological examinations of the hippocampus are shown in Figure 5A. Compared with those of mice in the MC group, the nuclei of some neurons in the DG and CA3 subregions of the hippocampus of mice in the MM group were deeply stained and contracted, the number of subregion cells was reduced, and the neurons in the CA1 subregion were not significantly damaged; there was no significant difference between the FC group and MC group. Compared with those of mice in the FC group, the nuclei of some neurons in the DG and CA3 subregions of the hippocampus of mice in the FM group were deeply stained and contracted, while there was no significant difference in the number of neurons in the CA1 subregion. Compared with those of mice in the MM group, the CA3 subregion of the hippocampus of mice in the FM group was less damaged.

### 3.6 Effects of Stress on the Rectal Tissue of Mice

The findings of rectal histopathological examinations are shown in Figure 5B. Compared with those in mice in the MC group, the cell nuclei in the transverse section of the rectum in the MM group were more condensed, the villus cells were destroyed in the longitudinal section, and there was diffuse ulceration and inflammatory cell infiltration. The rectal tissue cells of mice in the FC group were not significantly different from those of mice in the MC group. Compared with those from mice in the FC group, the nuclei in longitudinal rectal sections from mice in the FM group were pyretic, and local ulceration and inflammatory cell infiltration were observed. Compared with those from mice in the MM group, there was less inflammatory cell infiltration and local ulceration in rectal intestinal tissue from mice in the FM group.

### 3.7 Effects of Stress on the Gut Microbiota in Mice

#### 3.7.1 Diversity of the Gut Microbiota

**FIGURE 6:**
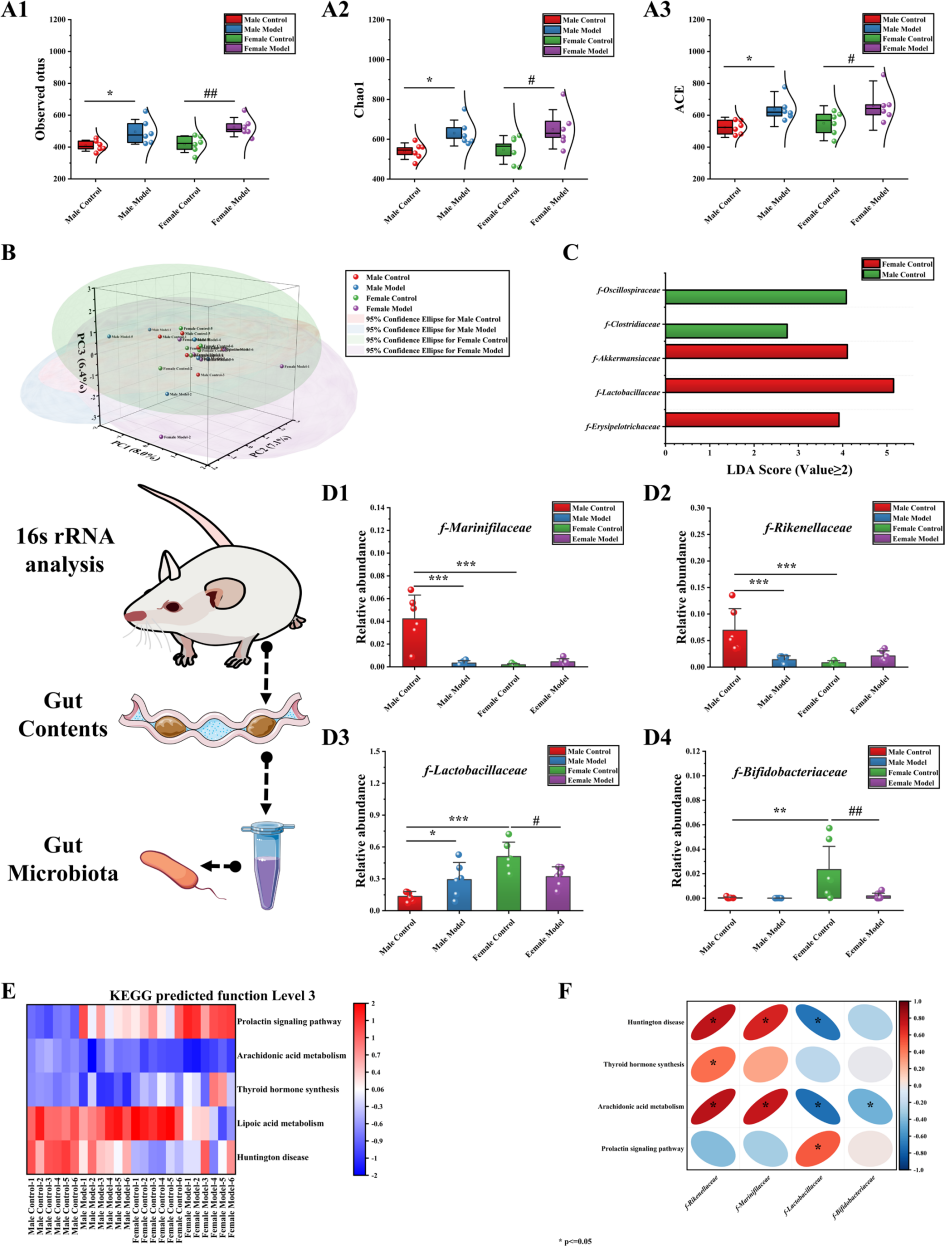
Effects of stress on gut microbiota in mice. (A) α diversity analysis, (B) β diversity analysis, (C) LEfSe analysis, (D) differences in the gut microbiota at the family level, (D) level 3 KEGG pathway function prediction, and (E) Spearman correlation analysis. The data are shown as the means ± SDs (*n* = 6). *p < 0.05, **p < 0.01, and ***p < 0.001 denote significant differences from the MC group. ^#^ *p* < 0.05 and ^##^ *p* < 0.01 denote significant differences from the FM group.

According to the α diversity analysis (Figure 6A), the number of observed OTUs (*p* < 0.05), Chao1 index (*p* < 0.05), and ACE index (*p* < 0.05) in mice in the MM group were significantly different from those of mice in the MC group. Compared with those of mice in the FC group, the number of observed OTUs (*p* < 0.01), Chao1 index (*p* < 0.05), and ACE index (*p* < 0.05) of mice in the FM group were significantly different. Principal coordinate analysis (PCA) of β diversity based on Bray–Curtis distances revealed differences in microbiota composition and structure among the MC, MM, FC, and FM groups (PC1: 8.0%, PC2: 7.1%, PC3: 6.4%) (Figure 6B). As shown in Figure 6C, linear discriminant analysis effect size (Lefse) analysis revealed two species (*f‐Oscillospiraceae* and *f‐Clostridiaceae*) and 3 species (*f‐Akkermansiaceae*, *f‐Lactobacillaceae*, and *f‐Erysipelotrichaceae*) of gut microbiota with linear discriminant analysis (LDA) values greater than 2 in the MC group and FC group, respectively.

#### 3.7.2 Differences Among Groups of Gut Microbiota at the Family Level

The results of bacterial community analysis at the family level are shown in Figure 6D. Compared with those of mice in the MC group, the abundances of *f‐Lactobacillaceae* (*p* < 0.05) in the MM group were significantly increased, whereas those of *f‐Marinifilaceae* (*p* < 0.001) and *f‐Rikenellaceae* (*p* < 0.001) were significantly decreased. Compared with those of mice in the FC group, the abundances of *f‐Lactobacillaceae* (*p* < 0.05) and *f‐Bifidobacteriaceae* (*p* < 0.01) in the FM group were significantly decreased. Compared with those of mice in the MC group, the abundances of *f‐Lactobacillaceae* (*p* < 0.001) and f*‐Bifidobacteriaceae* (*p* < 0.01) in the FC group were significantly increased, whereas those of *f‐Marinifilaceae* (*p* < 0.001) and *f‐Rikenellaceae* (*p* < 0.001) were significantly decreased.

#### 3.7.3 PICRUSt2 Level‐3 KEGG Pathway Functional Prediction and Associated Gut Microbiota

There were five different pathways in the level‐3 KEGG pathway functional prediction (Figure 6E), including the Huntington disease, lipoic acid metabolism, thyroid hormone synthesis, arachidonic acid metabolism, and prolactin signaling pathways. Compared with that in the MC group, the expression of genes involved in Huntington disease, lipoic acid metabolism, thyroid hormone synthesis, and arachidonic acid metabolism pathways was significantly downregulated, whereas the expression of genes involved in the prolactin signaling pathway was significantly upregulated. Compared with that in the FC group, the expression of genes involved in Huntington disease, the prolactin signaling pathway, thyroid hormone synthesis pathway, and arachidonic acid metabolism pathway was significantly upregulated, whereas the expression of genes involved in the lipoic acid metabolism pathway was significantly downregulated. Compared with that in the MM group, the expression of genes involved in Huntington disease, lipoic acid metabolism, and arachidonic acid metabolism pathways in the FM group was significantly upregulated, while the expression of genes involved in thyroid hormone synthesis and prolactin‐signaling pathways was significantly downregulated.

Spearman correlation analysis was performed on the composition of different gut microbiota and the level‐3 KEGG pathway functional prediction, and the results are shown in Figure 6F. Huntington disease was positively correlated with *f‐Marinifilaceae* (*p* < 0.05) and *f‐Rikenellaceae* (*p* < 0.05) and negatively correlated with *f‐Lactobacillaceae* (*p* < 0.05). Thyroid hormone synthesis was positively correlated with *f‐Rikenellaceae* (*p* < 0.05). The proline‐signaling pathway was positively correlated with *f‐Lactobacillaceae* (*p* < 0.05). Arachidonic acid metabolism was positively correlated with *f‐Marinifilaceae* (*p* < 0.05) and *f‐Rikenellaceae* (*p* < 0.05), whereas *f‐Lactobacillaceae* (*p* < 0.05) and *f‐Bifidobacteriaceae* (*p* < 0.05) were negatively correlated.

### 3.8 Effects of Stress on Gut Metabolism in Mice

**FIGURE 7:**
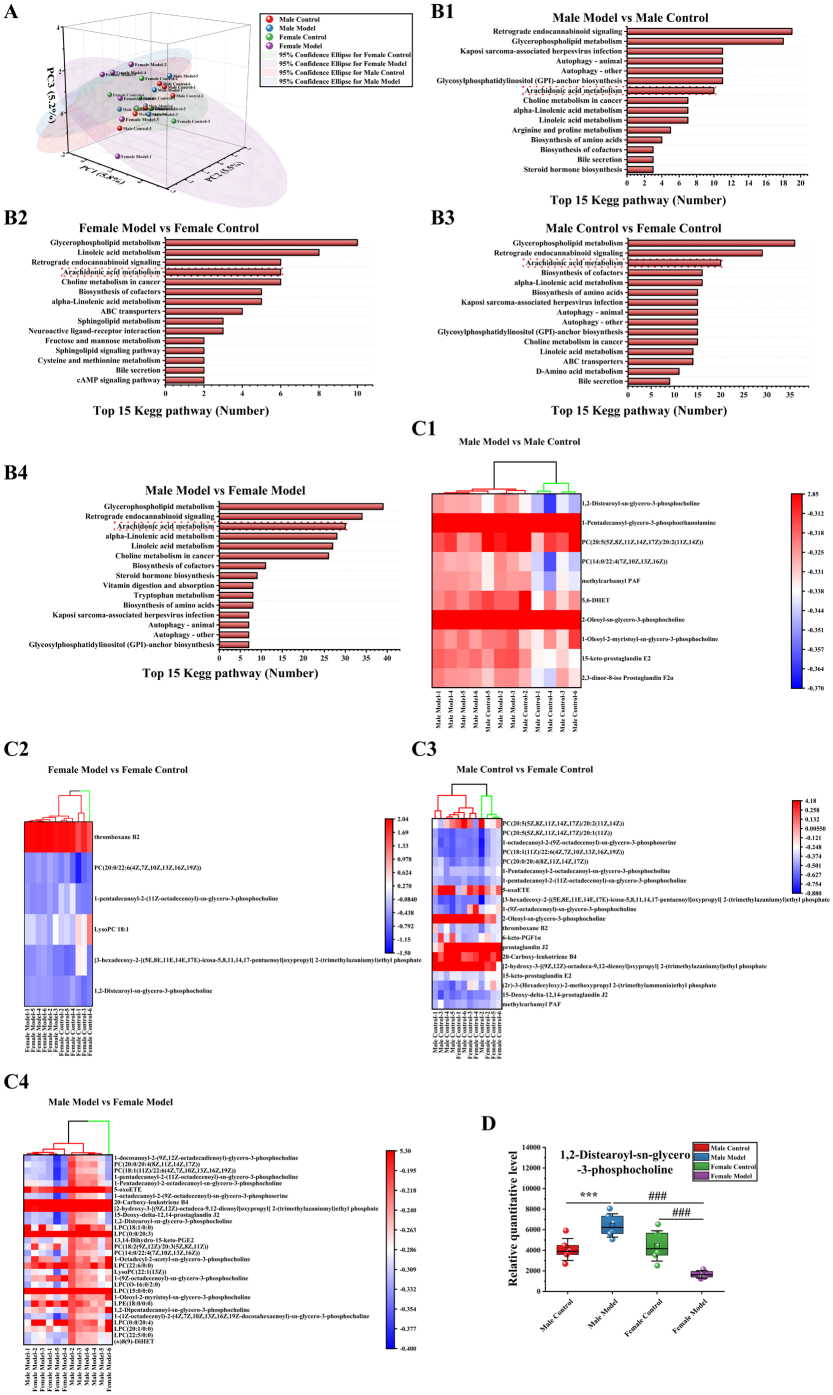
Effects of stress on gut metabolism in mice. (A) PCA, (B) top 15 KEGG pathways, (C) differentially abundant metabolite clustering heatmap, (D) relative quantification of metabolites. The data are shown as the means ± SDs (*n* = 6). ***p < 0.001 compared with the MC group; ^###^ *p* < 0.001 compared with the FM group.

PCA revealed differences in metabolic composition among the mouse groups (PC1: 5.8%, PC2: 5.5%, PC3: 5.2%) (Figure 7A). The KEGG pathway for differential metabolite (DM) enrichment in mice in each group was the same as the bacterial function prediction pathway, which is the arachidonic acid metabolism pathway (Figure 7B). As shown in Figure 7C, cluster analysis was performed on the DMs of the arachidonic acid metabolism pathway. In the MM group vs. the MC group, 2 DMs were upregulated, and 8 DMs were downregulated; in the FM group vs. the FC group, 1 DM was upregulated, and 5 DMs were downregulated; in the FC vs. the MC group, 4 DMs were upregulated, and 16 DMs were downregulated; and in the FM vs. the MM group, 17 DMs were upregulated, and 13 DMs were downregulated.

There was a common DM (G‐DSGP: gut‐1,2‐distearoyl‐sn‐glycero‐3‐phosphocholine) (Figure 7D) in the arachidonic acid metabolism pathway in all groups. Compared with that of mice in the MC group, the level of G‐DSGP in mice in the MM group was significantly increased (*p* < 0.001). Compared with that of mice in the FC group, the level of G‐DSGP in mice in the FM group was significantly decreased (*p* < 0.001). Compared with that of mice in the MM group, the level of G‐DSGP in mice in the FM group was significantly decreased (*p* < 0.001).

### 3.9 Effects of Stress on Hippocampal Metabolism in Mice

**FIGURE 8:**
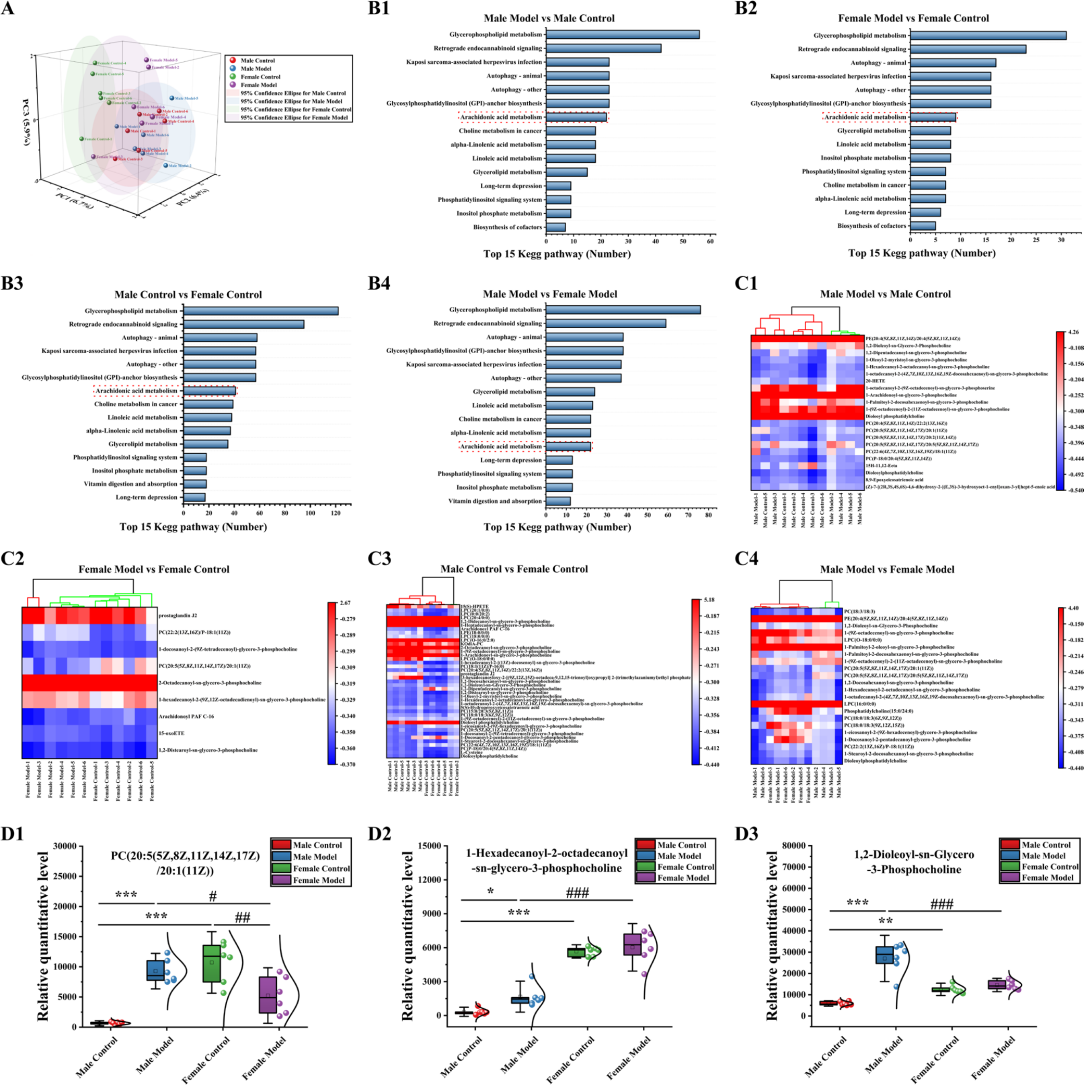
Effects of stress on hippocampal metabolism in mice. (A) PCA, (B) top 15 KEGG pathways, (C) differentially abundant metabolite clustering heatmap, (D) relative quantification of metabolites. The data are shown as the means ± SDs (*n* = 6). *p < 0.05, **p < 0.01, and ***p < 0.001 compared with the MC group; ^#^ *p* < 0.05, ^##^ *p* < 0.01, and ^###^ *p* < 0.001 compared with the FM group.

PCA revealed differences in metabolic composition among the mouse groups (PC1: 6.7%, PC2: 6.4%, PC3: 5.9%) (Figure 8A). The KEGG pathway for DM enrichment in mice in each group was the same as that for the intestinal metabolic pathway and the microbial function prediction pathway, which was the arachidonic acid metabolism pathway (Figure 8B). As shown in Figure 8C, cluster analysis of DMs in the arachidonic acid metabolism pathway revealed that 19 DMs were upregulated, and 3 DMs were downregulated in the MM vs. MC group. In the FM group vs. FC group, 6 DMs were upregulated, and 3 DMs were downregulated. In the FC vs. MC group, 21 DMs were upregulated, and 20 DMs were downregulated. In the FM vs. MM group, 12 DMs were upregulated and 10 DMs were downregulated.

There were three common DMs (PC‐20: PC(20:5(5Z, 8Z, 11Z, 14Z, 17Z)/20:1(11Z)), HOSGP: 1‐hexadecanoyl‐2‐octadecanoyl‐sn‐glycero‐3‐phosphocholine, H‐DSGP: hippocampus‐ 1,2‐dioleoyl‐sn‐glycero‐3‐phosphocholine) in the arachidonic acid metabolism pathway in each group (Figure 8D). Compared with those of mice in the MC group, the levels of PC‐20 (*p* < 0.001), HOSGP (*p* < 0.05), and H‐DSGP (*p* < 0.001) of mice in the MM group were significantly increased. Compared with that of mice in the FC group, the level of PC‐20 (*p* < 0.01) of mice in the FM group was significantly decreased. Compared with those of mice in the MC group, the levels of PC‐20 (*p* < 0.001), HOSGP (*p* < 0.001), and H‐DSGP (*p* < 0.01) of mice in the FC group were significantly increased. Compared with that of mice in the MM group, the level of HOSGP (*p* < 0.001) of mice in the FM group was significantly increased, whereas the levels of PC‐20 (*p* < 0.05) and H‐DSGP (*p* < 0.001) were significantly decreased.

### 3.10 Spearman Correlation Analysis

#### 3.10.1 Association Analysis Was Performed Between Behavior and Biological Indicators

**FIGURE 9:**
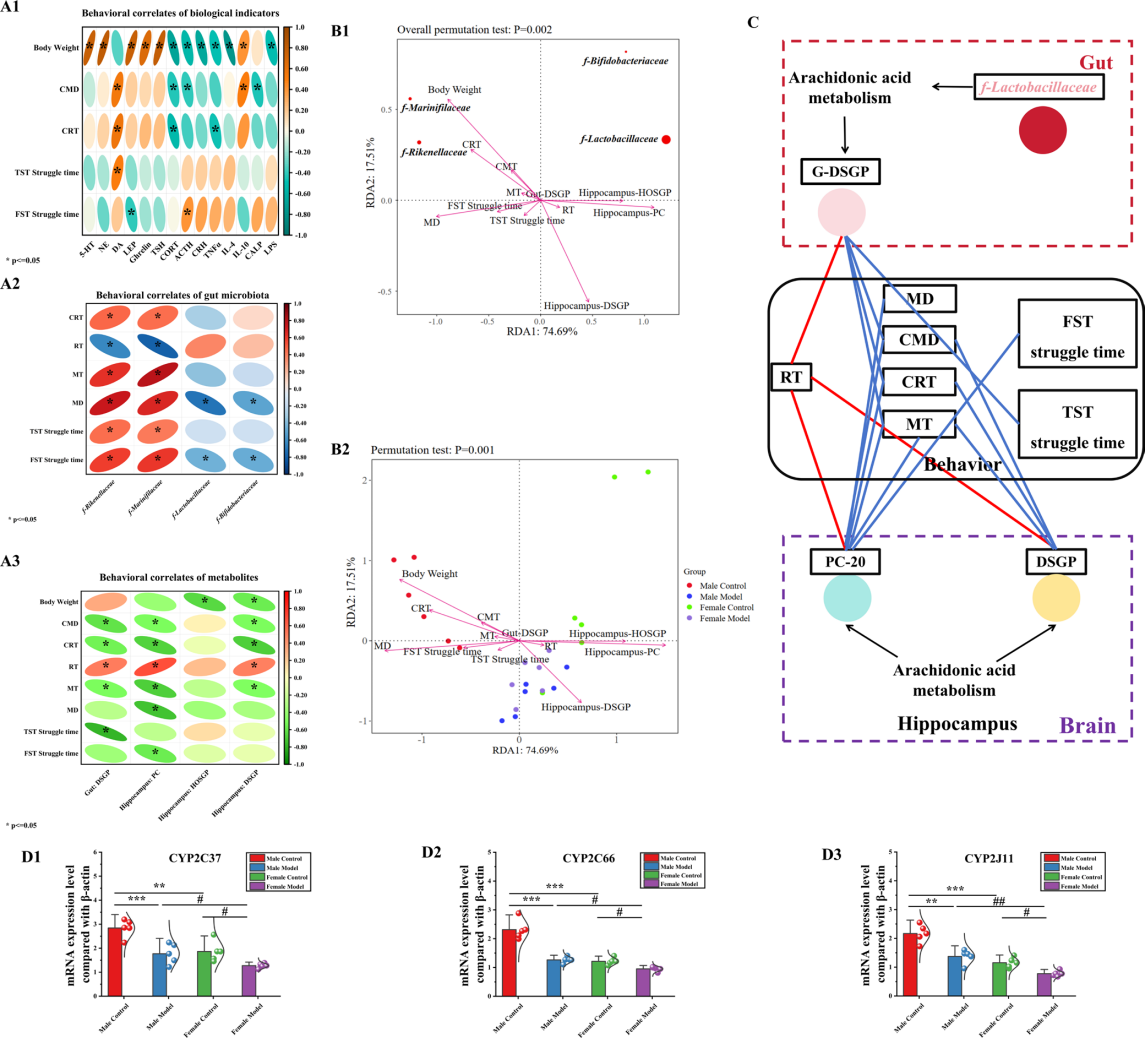
Multi‐omics association analysis and Q‐PCR verification of CYP enzyme system metabolism. (A) Spearman correlation analysis, (B) RDA analysis, (C) network diagram, (D) q‐PCR verification. In (A) and (B), *n* = 6; in (D), the data are shown as mean ± SDs (*n* = 5). *p < 0.05, **p < 0.01, and ***p < 0.001 compared with the MC group; ^#^ *p* < 0.05 and ^##^ *p* < 0.01 compared with the FM group. In Panel C, the line color represents the correlation, red represents a positive correlation, and blue represents a negative correlation.

The associations between behavior and biological indicators are shown in Figure 9A3. Body weight was significantly positively correlated with monoamine neurotransmitter (5‐HT: *p* < 0.05; NE: *p* < 0.05), appetite indicator (ghrelin: *p* < 0.05; LEP: *p* < 0.05; TSH: *p* < 0.05), and anti‐inflammatory factor (IL‐10: *p* < 0.05) levels and significantly negatively correlated with pro‐inflammatory factor (TNFα: *p* < 0.05; IL‐4: *p* < 0.05; LPS: *p* < 0.05) and HPA axis indicator (CORT: *p* < 0.05; ACTH: *p* < 0.05; CRH: *p* < 0.05) levels. The CRT level was significantly positively correlated with DA (*p* < 0.05) and IL‐10 (*p* < 0.05) levels and significantly negatively correlated with CORT (*p* < 0.05), ACTH (*p* < 0.05), and CALP (*p* < 0.05) levels. The CMD was significantly positively correlated with the DA level (*p* < 0.05) and significantly negatively correlated with CORT (*p* < 0.05) and TNFα (*p* < 0.05) levels. TST struggling time was significantly positively correlated with the DA level (*p* < 0.05). FST struggling time was significantly positively correlated with ACTH (*p* < 0.05) and LEP (*p* < 0.05) levels.

#### 3.10.2 Association Analysis Was Performed Between Behavior and Gut Microbiota

The associations between behavior and gut microbiota are shown in Figure 9A2. *f‐Marinifilaceae* and *f‐Rikenellaceae* were significantly positively correlated with the CRT level (*p* < 0.05; *p* < 0.05), MT (*p* < 0.05; *p* < 0.05), MD (*p* < 0.05; *p* < 0.05), and TST (*p* < 0.05; *p* < 0.05) and FST struggle times (*p* < 0.05; *p* < 0.05) and significantly negatively correlated with the RT (*p* < 0.05; *p* < 0.05). The abundances of *f‐Lactobacillaceae* and *f‐Bifidobacteriaceae* were significantly negatively correlated with the MD (*p* < 0.05; *p* < 0.05) and the FST struggling time (*p* < 0.05; *p* < 0.05).

#### 3.10.3 Association Analysis Was Performed Between Behavior and Differential Metabolites

The associations between behavior and differential metabolites are shown in Figure 9A3. The G‐DSGP level was significantly positively correlated with the RT (*p* < 0.05) and significantly negatively correlated with the MT (*p* < 0.05), CRT level (*p* < 0.05), CMD (*p* < 0.05), and TST struggling time (*p* < 0.05). The hippocampal PC‐20 concentration was significantly positively correlated with the RT (*p* < 0.05) and significantly negatively correlated with the MT (*p* < 0.05), MD (*p* < 0.05), CRT level (*p* < 0.05), CMD (*p* < 0.05), and the FST struggling time (*p* < 0.05). There was a significant negative correlation between the hippocampal HOSGP level and body weight (*p* < 0.05). The G‐DSGP level was significantly positively correlated with the RT (*p* < 0.05) and significantly negatively correlated with the MT (*p* < 0.05), CRT level (*p* < 0.05), CMD (*p* < 0.05), and body weight (*p* < 0.05).

### 3.11 RDA Analysis

The RDA analysis was used to perform a dimensionality reduction analysis of the different gut flora, metabolites, and depressive behavior. The length of the arrow represents the intensity of the influence of the factor on the change in flora, and the longer the length, the greater the influence of environmental factors. As shown in Figure 9B1, the relationship between different gut flora and metabolites and depressive behavior is in the order of *f‐Lactobacillaceae* > *f‐Rikenellaceae* > *f‐Marinifilaceae* > *f‐Bifidobacteriacea*. The degree of influence of metabolites and depressive behavior on different microbiota was body weight >hippocampal PC >hippocampal DSGP > MD > CRT > CMT > hippocampal HOSGP > FST struggling time > TST struggling time > MT > gut DSGP. It can be concluded that body weight, hippocampal PC, and hippocampal DSGP were important factors influencing the differential flora.

### 3.12 q‐PCR Results of Cytochrome P450 (CYP) Enzyme on the Arachidonic Acid Metabolic Pathway

The results of q‐PCR are shown in Figure 9D. Compared with the MC group, CYP2C37 (37.75%, *p* < 0.001; 34.47%, *p* < 0.01), CYP2C66 (45.34%, *p* < 0.001; 47.52%, *p* < 0.001), and CYP2J11(36.73%, *p* < 0.01; 46.77%, *p* < 0.001) were significantly decreased. Compared with the FC group, the levels of CYP2C37 (31.65%, *p* < 0.05), CYP2C66 (21.65%, *p* < 0.05), and CYP2J11 (32.86%, *p* < 0.05) in the FM group were significantly decreased. Compared with the MM group, the levels of CYP2C37 (28.05%, *p* < 0.05), CYP2C66 (24.76%, *p* < 0.05), and CYP2J11 (43.51%, *p* < 0.01) in the FM group were significantly decreased.

## 4 Discussion

According to etiological studies, stress is the main cause of depression [35]. In life, individuals frequently experience constant stress caused by predictable events and stressors caused by unpredictable events, making the mixed stress model more relevant to real‐life circumstances. Therefore, our previous studies have explored the pathway by which CRS + CUMS mixed stress induces depression [27, 28]. In this study, we selected CRS + CUMS‐exposed mice as stress‐induced depression model animals to explore differences in performance following stress exposure between female and male mice. Weight change and loss of appetite are among the core manifestations of depression [36]. Therefore, after 14 days of stress, we examined the body weight and food consumption of the mice, and we found that the body weight and 2‐h food consumption of the female and male mice were significantly decreased. The weight of male mice decreased more significantly (M: 17.81% vs. F: 12.97%), whereas the 2‐h food consumption of female mice decreased more significantly (F: 27.09% vs. M: 20.97%). These results indicate that mixed stress has the greatest effect on body weight in male mice and the greatest effect on appetite in female mice. Behavior is the most intuitive manifestation of sex differences. We used the OFT to measure the activity of mice in a new environment [29] and the TST and FST [30, 31] to evaluate the ability of mice to struggle in a desperate environment. The results showed that female mice had lower activity time and fight time before stress. After 14 days of stress, the activity capacity and fight time of female mice increased, and the trend of change was opposite to that of male mice. These behavioral results suggest that stress stimuli make female mice more prone to anxiety‐like behavior, whereas male mice are prone to depression‐like behavior. It is well known that, due to differences between males and females, females are more docile and cautious [37], whereas males are relatively active and positive [38]. Under stress, women are more likely to become agitated and show anxiety‐like behavior [39], and Bahrami et al. [40] found that girls are more prone to anxiety, which is consistent with the behavior of female mice in our behavioral tests. However, males under stress are more likely to experience extreme emotions such as self‐harm and suicide [41, 42], so their ability to fight in desperate situations is reduced. In addition, the study found that there are sex differences in the neural mechanism of behavior regulation [34], so abnormal behavior caused by stress stimulation may also have sex differences. Therefore, we hypothesize that stress may cause women to be prone to anxiety and men to be prone to depression.

Monoamine neurotransmitter‐level decline [43], HPA axis hyperactivity [44], and inflammatory cytokine [45] upregulation are the main physiological pathways of depression, so we examined these indicators after the stressed mice exhibited depression‐like behavior. The test results showed that the levels of 5‐HT, NE, and the anti‐inflammatory factor IL‐10 were significantly decreased in both female and male mice after stress, and the levels of HPA axis core indices and inflammatory factors were also significantly increased, which was consistent with the changes in physiological indices after depression [46, 47]. Interestingly, however, female mice showed high levels of DA (7.09%) and IL‐10 (10.64%, *p* < 0.001) before stress, as well as lower levels of 5‐HT (−16.88%, *p* < 0.01), NE (−11.79%), and inflammatory cytokines (TNFα: −11.68%, *p* < 0.05; IL‐6: −14.74%, *p* < 0.01). In addition, the DA level of female mice increased after stress, while that of male mice decreased (M: −26.55% vs. F: 13.37%). Moreover, the DA level was positively correlated with the CMT (*p* < 0.05), CMD (*p* < 0.05), and TST struggling time (*p* < 0.05); these data suggest that the increase in DA level may be related to the activity performance and anxiety‐like behavior of female mice [48, 49] and that DA may be a potential biomarker for distinguishing between male and female stress. Stress also caused a decrease in the levels of appetite hormones in male and female mice, among which the levels of ghrelin (−5.23%) and TSH (−9.16%) decreased more in female mice than in male mice, which also explains why the 2‐h food consumption of female mice was lower than that of male mice.

Studies have shown that stress can also cause structural damage in the hippocampus and intestine of mice [50, 51]; therefore, we conducted pathological tests on the gut and cerebral hippocampal tissues of mice. The results showed that stress caused different degrees of damage to rectal tissue and hippocampal tissue in both female and male mice, which were the same as the results of Katsuki et al. [52] and Cheng et al. [53]. However, stress damages the CA3 subregion of the male hippocampus to a greater degree than it does in the female hippocampus, which may be related to differences in hippocampal function and structure between male and female mice [54]. In addition, the degree of rectal tissue damage caused by stress was greater in male mice than in female mice. We then tested indices related to gut damage [55] and found that after stress, the levels of LPS (43.61%, *p* < 0.001) and CALP (−8.00%, *p* < 0.05) in female mice were significantly lower than those in male mice. Studies have shown that men are prone to ulcerative bowel disease [56], possibly because adult women have stronger innate and adaptive immune responses than men [57]. According to these results, stress is more likely to cause structural damage to the hippocampus and intestines in male mice.

Stress is known to be closely associated with the gut microbiota [58, 59]. Previous studies have shown that there are sex differences in the gut microbiota, especially in key Phylum such as *Actinomycetes*, *Bacteroidetes*, and *Firmicutes* [60, 61]. We therefore examined the gut microbiota of both male and female mice. The results showed that male mice had a high level of *f‐Rikenellaceae* and a low level of *f‐Lactobacillaceae* before stress, whereas female mice had a high relative abundance of *f‐Lactobacillaceae* and *f‐Bifidobacteriaceae*. Existing studies have found that the intestinal *f‐Lactobacillaceae*, *f‐Rikenellaceae*, and *f‐Bifidobacteriaceae* are significantly decreased in patients with depression [62, 63, 64]. Our experiment found that after stress, the relative abundance of *f‐Rikenellaceae* decreased in male mice, whereas the relative abundance of *f‐Bifidobacteriaceae* and *f‐Lactobacillaceae* decreased in female mice, and the change trend of *f‐Lactobacillaceae* was opposite to that of male mice. These results may reflect the difference in the microbiota of male and female mice during stress, which may be one of the reasons for the different abnormal behaviors of male and female mice. In addition, *Lactobacillaceae* and *Bifidobacteriaceae* are probiotics that play an important role in improving general immunity, gastrointestinal infections, and depressive symptoms [65, 66, 67]. However, compared to male mice, female mice had higher levels of *f‐Lactobacillaceae* and *f‐Bifidobacteriaceae* before and after stress. These results suggest that female mice may have a greater ability to immunomodulate, which is consistent with the results above showing lower levels of inflammation and less damage to intestinal structures in female mice. Brivio et al. also found that male mice are more sensitive to chronic mild stress [68]. Therefore, in combination with the experimental results of this study, we speculated that female mice may regulate their immune ability through the flora, and their stress resistance may be stronger than that of male mice.

Gut microbiota can influence host behavior by directly regulating metabolic levels, producing neuroactive substances and short‐chain fatty acids [69, 70]. We found differences in AA (arachidonic acid) metabolism between female and male mice via functional prediction of the gut microbiota. After stress, AA metabolism in male mice was significantly downregulated (*p* < 0.05), while the regulation trend of AA metabolism in female mice was opposite to that in male mice. AA metabolism was correlated with different bacterial populations. In our next metabolomic examination of the gut and hippocampus, metabolites involved in AA metabolism were found to be enriched, which was consistent with the prediction of microbiota function. AA is an important polyunsaturated fatty acid present in mammalian tissues and is usually esterified as glycerolipids or glycerolipids to maintain the structure and function of cell membranes [71]. AA metabolism is involved in diseases such as those of the nervous system [72, 73] and cardiovascular system [74, 75], as well as immune and inflammatory reactions in vivo [76, 77]. Richard found that in bipolar disorder, the turnover of AA and its metabolites was reduced [78], and the metabolic network of AA was the main network for generating inflammatory mediators and inducing inflammation [79]. Phosphatidylcholine (PC) is a metabolite of AA [80]. PC is involved in anti‐inflammatory activity and the regulation of systemic immunity [81, 82]. Our results revealed significant sex differences in the levels of G‐DSGP in the gut and PC‐20 in the hippocampus before stress, and females showed high levels of G‐DSGP and PC‐20, which may be related to the stronger immunity of females. However, the levels of G‐DSGP and PC‐20 decreased in female mice after stress, while they increased in male mice. At present, there is a lack of specific research on G‐DSGP and PC‐20, and we know only that PC can regulate systemic immunity [81, 82]. We speculate that stress causes high levels of inflammation in males, and males are more sensitive to stress [68], which promotes the metabolism of some types of PC and increases the adaptability of the body. Females are less affected by stress, and PC metabolism may be at a basal level. It may also be that G‐DSGP and PC‐20 levels are related to sex differences caused by stress. Although further studies are needed to understand the role of G‐DSGP and PC‐20 in regulating inflammatory function, there are sex differences between G‐DSGP and PC‐20 in mice before and after stress, which provides some directions for future research. As AA metabolism may play an important role in the differential process of male and female stress, CYP can metabolize arachidonic acid to hydroxy‐eicosatetraenoic acid and epoxy‐eicosatetraenoic acid. These AAs are formed in a tissue‐ and cell‐specific manner and have many biological functions. In addition, CYP‐derived arachidonic acids are also important in the regulation of ion transport and have recently been shown to influence many fundamental biological processes, including cell proliferation, apoptosis, inflammation, and hemostasis. The formation of these functionally related arachidonic acids is tightly controlled by the expression and activity of CYP cyclooxygenase and hydroxylase [38]. Therefore, we further detected the expression of key enzymes in the CYP enzyme metabolic system by q‐PCR. Our results showed that before stress, there were significant differences in CYP enzymes (CYP2C37, CYP2C66, and CYP2J11) in the female control group compared to the male control group mice. In addition, stress caused a significant decrease in the expression of CYP2C37, CYP2C66, and CYP2J11 in the hippocampus of male and female mice. The decrease fluctuation of CYP enzyme (CYP2C37, CYP2C66, and CYP2J11) was greater in male model mice, which was significantly higher than that in female model mice. This may be related to differences in the expression of metabolites in the AA pathway. Studies have shown that the CYP2C and CYP2J gene subfamilies are involved in immune regulation during LPS‐induced inflammation [83], and the expression of the CYP2C and CYP2J gene subfamilies in mouse tissues has also been shown to have sex differences [34]. Therefore, we speculated that the AA pathway plays an important role in the stress process of male and female mice, and the different stress behavior may be caused by different levels of immune regulation.

We then attempted to use association analysis to perform Spearman correlation analysis between behavioral indices and levels of monoamine neurotransmitters, appetite indicators, and immune inflammatory factors, as well as differential gut flora and metabolites, and to construct association networks (Figure 9C). These results suggest that stress induces significant sex differences in monoamine neurotransmitter and appetite indicator levels, as well as inflammatory responses, in female and male mice. There were also differences in gut microbiota changes (*f‐Rikenellaceae*, *f‐Bifidobacteriaceae*, and *f‐Lactobacillaceae*) and AA metabolic pathways (G‐DSGP and PC‐20). Next, we analyzed the dimensions used to reduce different gut microbiota, metabolites, and depressive behavior by RDA. The results showed that *f‐Lactobacillaceae* were most affected by metabolites and behavior, and body weight, hippocampal PC, and hippocampal DSGP were important factors influencing differences in gut microbiota. The results showed that F‐lactic acid bacteria were most affected by metabolites and behavior, and body weight, hippocampal PC, and hippocampal DSGP were important factors influencing differences in gut microbiota.

These differences may explain why stress causes females to show more anxiety‐like behaviors and males to show more depression‐like behaviors. In particular, DA levels, gut microbiota (*f‐Lactobacillaceae*), and metabolite levels (G‐DSGP and PC‐20) of male and female mice showed opposite trends. Our study is the first to consider sex differences in stress response through the pathway of brain–gut metabolic regulation, providing some basic research on the different pathogenic mechanisms caused by sex factors in the stress process, and some basis for considering sex factors in the potential sex‐specific treatment and diagnosis of depression in the future. However, due to the lack of sufficient clinical studies and human samples, this study is still limited, and more human experiments on brain‐gut metabolism are needed to verify the differences in stress caused by gender factors. Notably, it has been suggested that gender differences among researchers may influence stress responses in laboratory animals through unconscious behavioral cues [84]. In this study, the sex of the experimenter was effectively controlled as a potentially confounding variable by using a single‐sex experimenter to complete all feeding and behavioral operations. However, it should be pointed out that the mechanism of interaction between researchers of different genders and the stress response of male and female mice is still an important direction to be explored in this field. Therefore, future studies need to systematically verify the causal relationship between researcher gender and physiological and psychological indicators of experimental animals through multicenter, large‐sample experimental design.

## 5 Conclusion

Our results suggest that stress induces anxiety‐like behaviors in female mice and depression‐like behaviors in male mice. The sex differences in behaviors may be related to monoamine neurotransmitters, HPA axis hyperactivity, inflammatory factors, the gut microbiota, and brain–gut metabolism. Notably, stress caused opposite trends in the levels of DA, abundance of *f‐Lactobacillaceae*, and levels of metabolites (G‐DSGP and PC‐20) in male and female mice. These findings may provide evidence for sex differences in stress‐induced psychiatric disorders in the future.

## Author Contributions

All authors contributed a substantial workload to the research and writing of this article. The work of all authors is as follows: H.B. and T.G. designed the experimental study of this article; Y.Q., H.C., J.G., and Q.W. conducted the overall experiment of this article; Y.Q., X.L., and H.B. analyzed the experimental data and wrote the manuscript; Y.Q., X.Z., L.W., H.B., and T.G. interpreted the experimental results and critically revised the manuscript. This article is the final draft that has been read and approved by all authors.

## Ethics Statement

All animals used in this experiment were in compliance with ARRIVE guidelines and were conducted in strict accordance with the National Institutes of Health Guidelines for the Care and Use of Laboratory Animals (NIH Publication 18 No. 8023, revised in 1978), approved by the Chinese Academy of Sciences and approved by the Northwest Plateau Institute of Biology Committee for use in animal experiments (lot number NWIPB20171106‐01).

## Consent

The authors have nothing to report.

## Conflicts of Interest

The authors declare no conflicts of interest.

## Supporting information


Appendix S1.

## Data Availability

The authors declare that all data supporting the findings of this study are available upon reasonable request. Raw read files (SRA) for 16s rRNA sequencing of gut bacteria were deposited into the National Center for Biotechnology Information Sequence database (BioProject: PRJNA1005772). As this data relates to our upcoming experiments and articles, it will not be accessible until September 1, 2025.
