# Supplementary material for: A Study of Sex Differences in the Biological Pathways of Stress Regulation in Mice
Source: CNS Neurosci Ther. 2025 May 14;31(5):e70433. doi: 10.1111/cns.70433 (PMC12076126; doi:10.1111/cns.70433)
Supplement: Supplementary file 1 — Appendix S1. [file CNS-31-e70433-s001.docx]

**Quantitative PCR (q-PCR) procedure**

1. **RNA extraction procedure**

(1) Take the sample out and melt it on ice, absorb 1ml Trizol into 1.5 ml RNase free EP tube, and cut about 20 mg sample into EP tube;

(2) Add 3 mm enzyme-free grinding beads, break and mix them with a tissue grinder, take them out and leave them on ice for 5 min;

(3) Add 200 ul chloroform, shake and mix for 30 s, stand on ice for 5 min, 12000 rpm, 4℃, centrifuge for 10 min;

(4) Extract the upper water phase 400 ul into the new 1.5 ml RNase free EP tube to avoid pumping the protein in the middle layer;

(5) Add 400 ul 4°C pre-cooled isopropyl alcohol, mix it upside down, and place it at -20°C for 20 min;

(6) Centrifuge at 12000 rpm for 10 min, control the temperature to 4℃, remove the supernatant;

(7) Add 1ml of 75% ethanol, mix it upside-down, centrifuge at 12000 rpm for 5 min, control the temperature to 4℃, and remove the supernatant;

(8) Repeat Step 7

(9) Dry at room temperature for 5-10 min, volatilize clean ethanol (put in a super clean table);

(10) Add 30-50 ul DEPC water according to the precipitation amount, and let it stand at room temperature for 5 min to dissolve the RNA.

**2. RNA detection results**

In this study, the RNA OD value was detected by an ultramicro nucleic acid protein analyzer (scandrop100) using the A260/A280 ratio.

**3. Reverse transcription (consumables used are still enzyme-free and sterile)**

(1) Reverse transcription operation is performed using FOREGENE reverse transcription kit (RT Easy TM II) and 20 uL reaction system is adopted.

**The RT system is formulated as follows:**

Total RNA 1000 ng

2×RT OR-Easy TM Mix 10 μl

RNase Free dH2O To 20 μl

**Transcriptional reaction conditions are as follows:**

42℃ 15 min

85℃ 5 min

4℃ N/A

After the reaction, cDNA was obtained and stored at -80℃ for later use.

**4. primer design**

| **Primer name** | **Primer sequences (5 'to3')** |
| --- | --- |
| β-actin-F | GTATGAAGGCTTTGGTCTC |
| β-actin-R | TTGGTCTCAAGTCAGTGTA |
| cyp2j11-F | GGGATCCAGCCGTGATCATAA |
| cyp2j11-R | AGCTAAACTGGGGCGTTTCA |
| cyp2c37-F | GATGGCAATCAACCATTGCAAAA |
| cyp2c37-R | CTTGTTGTCTCTGTCCCAGCA |
| cyp2c66-F | GATCATGGGGAGGAGTTTGCT |
| cyp2c66-R | GCTGAAAACAACCCCACATCT |

**Table S1: primer design**

**5. q-PCR detection**

**Fluorescent q-PCR procedures and systems:**

（1）Step1- 95 ℃-2 Min

（2）Step2- 95 ℃-15 s

（3）Step3- 58℃-30 s +plate read

（4）Go to step2, 39 cycles

（5）Melt curve analysis（60 ℃~ 95 ℃）。

| **Component** | **Volume** |
| --- | --- |
| SYBR qPCR SuperMix plus | 5 μl |
| 10pmol/μl (10 μM) Primer #1 | 0.5 μl |
| 10pmol/μl (10 μM) Primer #2 | 0.5 μl |
| cDNA | 4 μl |
| Total volume | 10 μl |

**Table S2. Composition of the qPCR reaction solution**

**6 Test results**

**6.1 q-PCR results of all gene primers**

All components were configured according to the above q-PCR reaction system, centrifuged on a PCR plate centrifuge at 6000 rpm at 4℃ for 30 s, and then amplified in a q-PCR instrument according to the above procedure.


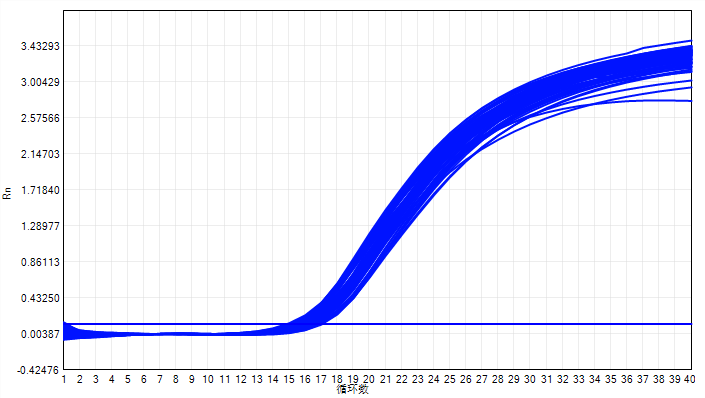

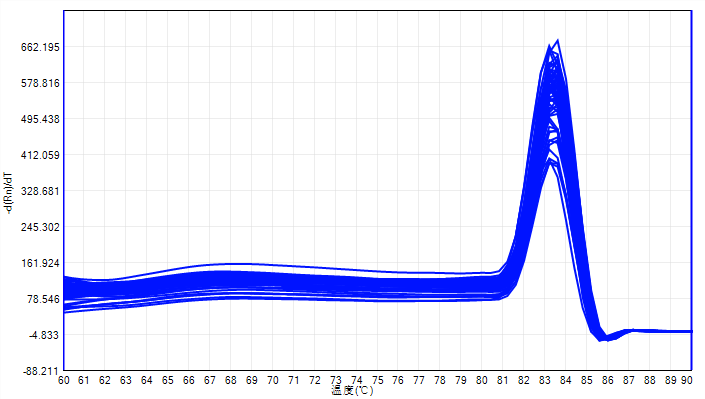


**Figure S1. The left figure is the amplification curve of β-actin, and the right figure is the dissolution curve**


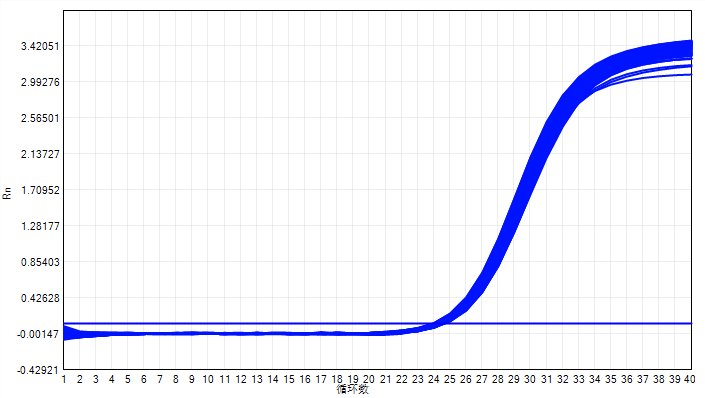

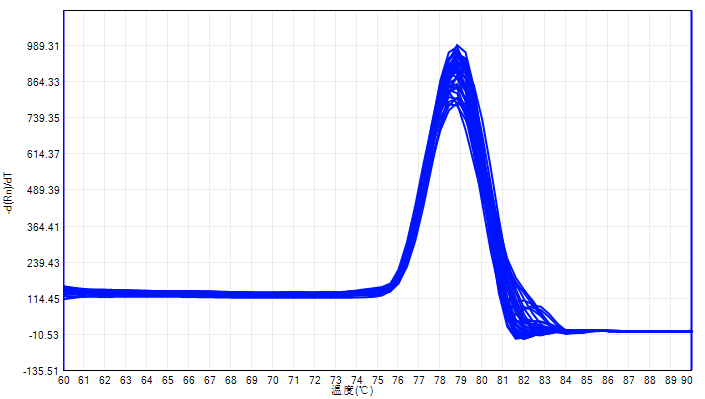


**Figure S1. The left figure shows the amplification curve of cyp2j11 and the right figure shows the dissolution curve**


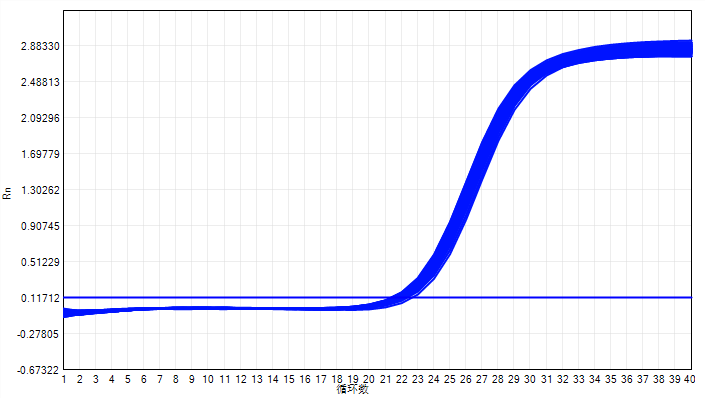

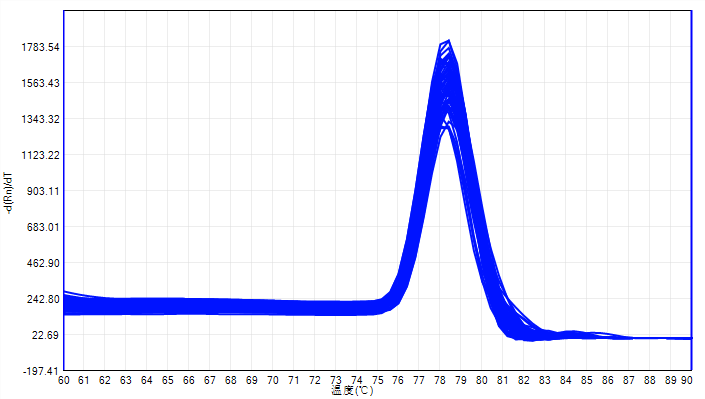


**Figure S1. The left figure shows the amplification curve of cyp2c37 gene, and the right figure shows the dissolution curve**


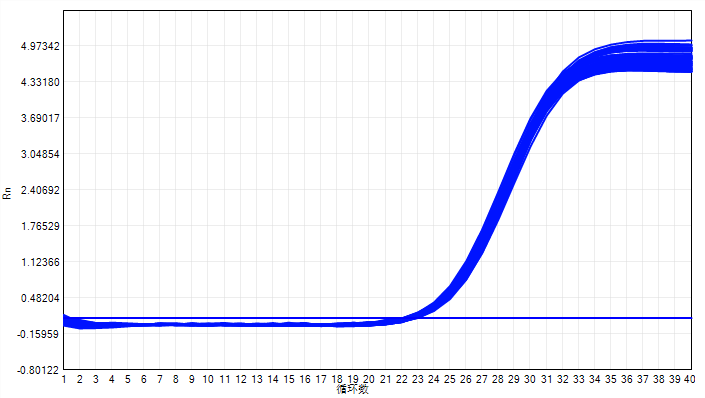

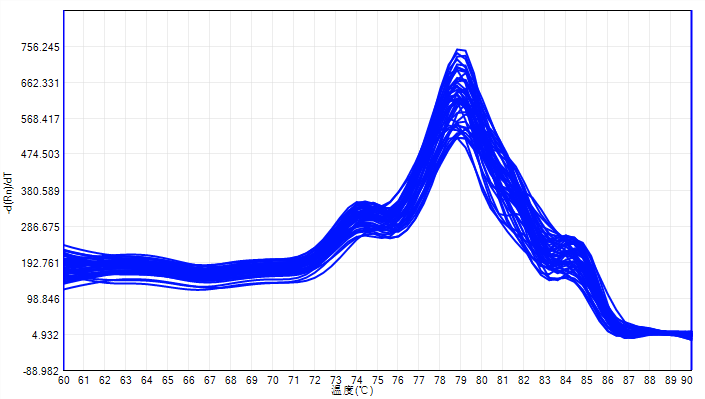


**Figure S2. The left figure shows the amplification curve of cyp2c66 gene, and the right figure shows the dissolution curve**

**6.2 Calculation of experimental results**

The relative expression of target genes in each sample was derived through the instrument software and calculated manually.

**The specific calculation method is as follows:**

ΔΔCT method:

ΔCT=CT (target gene, sample) -CT (internal reference gene, sample)

ΔΔCT=△CT (sample to be tested) - △CT (control sample)

Fold of expression =2^- ΔΔ CT^

Using β-actin as the internal reference, 2^^-△△CT^was used to calculate the relative gene expression of each sample.
